# Decreased circulating dipeptidyl peptidase-4 activity after short-term intensive insulin therapy predicts clinical outcomes in patients with newly diagnosed type 2 diabetes

**DOI:** 10.3389/fendo.2024.1352002

**Published:** 2024-02-27

**Authors:** Jie Chen, Peiji Dai, Weijian Ke, Xuesi Wan, Juan Liu, Lijuan Xu, Haipeng Xiao, Yanbing Li, Liehua Liu

**Affiliations:** Department of Endocrinology and Diabetes Center, The First Affiliated Hospital of Sun Yat-Sen University, Guangzhou, Guangdong, China

**Keywords:** type 2 diabetes mellitus, dipeptidyl peptidase 4, intensive insulin therapy, beta cell function, insulin resistance

## Abstract

**Background:**

This study aims to investigate the changes in circulating dipeptidyl peptidase-4 (DPP-4) activity following short-term intensive insulin therapy (SIIT) in newly diagnosed type 2 diabetes (T2D) patients and to assess its potential in predicting long-term remission.

**Methods:**

Ninety-five patients underwent SIIT for 2-3 weeks to attain and sustain near-normal glycemia. Insulin was then discontinued, and patients were followed for a year to evaluate glycemic outcomes. Biochemical tests, serum DPP-4 activity, and mixed meal tolerance tests were conducted at baseline, post-SIIT, and the 3-month follow-up.

**Results:**

DPP-4 activity decreased from 44.08 ± 9.58 to 40.53 ± 8.83 nmol/min/mL after SIIT (P<0.001). After three months post-SIIT, DPP-4 activity remained stable in the remission group (39.63 ± 8.53 nmol/L) but increased in the non-remission group (42.34 ± 6.64 nmol/L). This resulted in a more pronounced decrease in DPP-4 activity from baseline in the remission group (-3.39 ± 8.90 vs. -1.10 ± 8.95, P = 0.035). Logistic regression analyses showed that patients with greater DPP-4 activity reduction had a higher likelihood of 1-year remission (70% vs. 51.1%, OR: 7.939 [1.829, 34.467], P = 0.006 in the fully adjusted model). A non-linear relationship between △DPP-4 and 1-year remission rate was observed, with a clear threshold and saturation effect.

**Conclusion:**

Circulating DPP-4 activity significantly decreases after SIIT. The change in circulating DPP-4 activity during the 3-month post-treatment phase has the potential to predict long-term remission.

## 1 Introduction

Type 2 diabetes (T2D) has emerged as a critical public health issue, especially in developing countries like China. The prevalence of diabetes in Chinese adults is 12.4%, ranking first in the number of patients worldwide (1). Furthermore, over half of these patients have inadequate glycemic control. Longitudinal cohort studies have shown that achieving good glycemic control early, especially within the first year of diagnosis, can significantly reduce the risk of microvascular, macrovascular, and even mortality complications (2). Therefore, it is crucial to intervene in the various pathogenesis mechanisms of type 2 diabetes as soon as possible to attain sustained optimal glycemic control. Major mechanisms of T2DM include β-cell dysfunction and insulin resistance.

Recently, the reversal of T2D has attracted much research interest due to its ability to provide rapid and prolonged optimal glycemic control in the early stage. Many studies, including those from our center, showed that short-term intensive insulin therapy (SIIT) can induce long-term remission of diabetes in early-stage T2D patients (3–7). In previous studies, the one-year remission rate for newly diagnosed T2D patients after SIIT exceeded 50%, greatly facilitating long-term optimized glycemic control. This improvement was accompanied by improved insulin sensitivity and β-cell function. Based on this evidence, SIIT has now been recommended by the Chinese Diabetes Society as a standard therapy for patients with newly diagnosed T2D with remarkable hyperglycemia (8). However, although several predictors of treatment response have been identified in studies, there is currently no ideal method to predict long-term response after intensive treatment (3–6, 9, 10). This is particularly critical in the determination of subsequent management and whether to initiate drug treatment as early as possible, rather than observe until hyperglycemia relapse. Therefore, it is essential to explore new predictors of long-term remission.

Importantly, SIIT enhances insulin secretion not only in response to intravenous stimulation but also to oral challenge. This effect is crucial in reversing hyperglycemia (4, 11). As the incretin system plays an important role in postprandial glucose homeostasis, it’s essential to investigate the involvement of the incretin system component in the treatment response to SIIT. However, several studies have indicated that there is little to no alteration in fasting and postprandial GLP-1 levels following SIIT treatment (12, 13), whilst the role of DDP-4 remains to be determined.

DPP-4 is known for its ability to degrade incretins rapidly. In addition, DPP-4 is a multifunctional protein involved in various functions ranging from proteolysis, receptor co-stimulation of lymphocytes, adhesion, and immunity, to apoptosis (14). DPP-4 is expressed in a membrane-bound form on the surface of various cell types, including hepatocytes, lipocytes, gastrointestinal epithelial cells, endothelial cells, and lymphocytes. It is also present in the circulation in soluble form. DPP-4 in the circulation is thought to be secreted from the surface of DPP-4-expressing cells (15, 16). Apart from its capability to degrade incretins, circulating DPP-4 is also recognized as an adipokine associated with insulin resistance and T2D pathogenesis (17). Several studies have shown that circulating DPP-4 activity is significantly increased in T2D and is positively correlated with HbA1c levels (18–20). In a cohort study, increased circulating DPP-4 activity predicted the development of insulin resistance and the incidence of T2D in Chinese adults over 4 years (21). Hence, investigating changes in circulating DPP-4 before and after intensive treatment and its relationship to clinical response has significant clinical value, given little is known in this area.

Therefore, we investigated the changes in circulating DPP-4 activity caused by SIIT and its association with clinical outcomes. Furthermore, we explored its potential as a predictive factor for the therapeutic response to SIIT.

## 2 Methods

### 2.1 Participants

Participants were enrolled from the SIIT alone group of a randomized controlled trial (NCT00948324, ClinicalTrials.gov). This trial consisted of four groups. One group received SIIT alone, while the other three groups received sequential drug treatments after SIIT. As the purpose of this study was to investigate the relationship between DPP-4 activity and SIIT, we included SIIT alone group to exclude the influence of medication. A total of 95 patients of SIIT alone group were recruited to this research. 8 patients lost to follow-up before 12 months and 2 patients without available serum sample, and were thus excluded. The recruitment criteria of the original study were: no previous use of hypoglycemic agents, age between 25-70 years, body mass index (BMI) between 21-35 kg/m^2^, fasting blood glucose between 7.0-16.7 mmol/L, and no fertility plans. Exclusion criteria included diagnosis of diabetes other than T2D, acute or severe chronic diabetic complications, severe concomitant illnesses, use of medications known to affect blood glucose levels (for instance, systemic glucocorticoids), or the presence of severe concomitant diseases such as malignant tumors or chronic systemic inflammation.

### 2.2 Study design

The SIIT intervention for the participants was consistent with previous studies (22, 23). Briefly, all patients were hospitalized after diagnosis. Baseline assessments were performed after a run-in period of 2 to 3 days. Subsequently, SIIT was administered to each patient using an insulin pump (Medtronic Inc, Minneapolis, Minnesota, USA). Insulin lispro (Humalog, Eli Lilly and Company, Indianapolis, Indiana, USA) or Aspart (Novo Nordisk, Bagsværd, Denmark) was administered at an initial total daily dose of 0.5 IU/kg, where 50% was allocated as basal insulin, and the remaining 50% was divided into three equal premeal boluses. The insulin dose was titrated based on daily 8-point finger stick blood glucose values (before and 2 hours after each meal, before bedtime, and at 3 a.m.) to achieve glycemic targets (< 6.1 mmol/L for fasting/premeal blood glucose, and < 8.0 mmol/L for 2 hours postprandial blood glucose). During the hospitalization, daily caloric intake was suggested by a nutritionist, with carbohydrate, protein, and fat comprising 50-60%, 10-15%, and 20-30% of the total calories, respectively. Patients were encouraged to walk or jog for 0.5 hours after each meal to promote postprandial glycemic control. SIIT was maintained for 14 days until blood glucose targets were achieved. Insulin was stopped after the last pre-dinner bolus, and baseline measurements were repeated the next day (at least 15 hours after insulin withdrawal).

participants were followed up for 1 year after discharge. Lifestyle modifications and self-monitoring of capillary blood glucose were suggested to be maintained according to local diabetes guidelines. Glycemic remission was defined as fasting blood glucose < 7.0 mmol/L and glycated hemoglobin A1c (HbA1c) < 6.5% (48 mmol/mol) without anti-diabetic agents. If hyperglycemia recurred and was confirmed by venous blood glucose 1 week later, the standard diabetes treatment was initiated.

### 2.3 Blood sampling and measurements

Clinical assessments were performed before SIIT, after SIIT, and at 3-month follow-up. Anthropometric data such as height, body weight, and waist circumference were collected. Fasting venous blood samples were collected to measure liver enzymes, lipid profiles, fasting plasma glucose (FPG), HbA1c, high sensitivity C reactive protein (Hs-CRP), free fatty acid (FFA), and DPP-4 activity. A mixed meal tolerance test (MMTT) was conducted following an overnight fast via a 400 kcal instant noodles consumption. Blood samples were collected immediately before, at 30, 60, and 120 minutes after the meal.

Insulin sensitivity and beta cell function indices were derived from glucose and insulin level measurements during MMTT. The polygonal formula was used to calculate the area under the curve (AUC) of glucose and insulin. Homeostasis model assessment for β-cell function (HOMA-B) and insulin resistance (HOMA-IR) were calculated by 20×fasting insulin (FINS)/(FPG-3.5) and (FPG×FINS)/22.5, respectively. The Matsuda index was calculated using the formula: 10000/square root of [(FPG×FINS)×(mean glucose MMTT×mean insulin MMTT)]. Insulin Secretion-Sensitivity Index-2 (ISSI-2) was calculated as AUC insulin/AUC glucose×Matsuda index (24).

All clinical measurements were performed at the Central Clinical Laboratory of the First Affiliated Hospital of Sun Yat-Sen University. DPP-4 activity was assessed as previously reported (25), using Gly-Pro-p-nitroanilide as the substrate (Sigma, Poole, Dorset, UK). In brief, a 20 μL serum sample was incubated with 100 μL of 1 mmol/L Gly-Pro p-nitroaniline at 37°C for 60 minutes. The liberation of p-nitroaniline was then monitored at 405 nm using 1 mmol/L nitroaniline used as the reference standard.

### 2.4 Statistical analysis

Normally distributed data were expressed as mean ± standard deviation, whereas non-normally distributed data were shown as median values with interquartile range. Comparisons between normally distributed and non-normally distributed data were conducted using Student’s t-test and Wilcoxon’s signed-rank test, respectively. ANCOVA was applied to adjust covariates. Correlations between variables were analyzed using partial correlation analysis. Multiple linear regression analyses were performed when necessary. Kaplan-Meier curves were utilized to summarize time-to-event data. Factors predicting remission was identified through logistic regression model analyses. Smooth curve fitting was used to explore potential nonlinear relationships, with inflection points calculated using a two-piecewise linear regression model. All statistical procedures were performed using EmpowerStats (version 4.0, X&Y Solutions, Boston, MA, USA), R Software (version 4.2.0, R Foundation for Statistical Computing, Vienna, Austria), and GraphPad Prism version 9.0 (GraphPad, La Jolla, California, USA).

## 3 Results

### 3.1 Changes in circulating DPP-4 activity after SIIT treatment

The patients consisted of 77 (81.05%) males and 18 females, with a mean age of 46.85 ± 11.26 years and diabetes duration of 1 [interquartile range (IQR) 4.5] months. Similar to previous studies, BMI, cholesterol, triglycerides, low-density lipoprotein cholesterol (LDL-C), FPG, 2H-PG, fasting insulin, HOMA-IR, and AUC of glucose were all significantly decreased after SIIT. In contrast, HOMA-B, AUC of insulin, Matsuda index, and ISSI-2 were significantly increased. At 3 months, BMI and HbA1c continued to decrease while HOMA-B, AUC of insulin, and high-density lipoprotein cholesterol (HDL-C) continued to increase, compared with discharge (**Table 1**). Mean circulating DPP-4 activity decreased from 44.08 ± 9.58 nmol/min/mL at baseline to 40.53 ± 8.83 nmol/min/mL at discharge and was at 40.71 ± 9.05 nmol/min/mL at 3 months (P < 0.01 compared with baseline and P> 0.05 compared with after SIIT, respectively).

**Table 1:** Clinical parameters at baseline, after SIIT and at 3-month follow-up.

	Baseline	After SIIT	3-month
Diabetes duration (months)	1 (4.5)	–	–
Body weight (kg)	73.14 ± 12.21	71.92 ± 12.09^***^	69.75 ± 11.57^***###^
BMI (kg/m2)	25.94 ± 2.88	25.47 ± 2.89^***^	24.79 ± 2.99^***###^
Waist circumference (cm)	92.17 ± 8.47	91.29 ± 8.47^*^	87.97 ± 7.46^***###^
WHR	0.93 ± 0.06	0.93 ± 0.06	0.91 ± 0.05^***###^
ALT (U/L)	24 (22)	24.5 (15)	22 (15.5)
AST (U/L)	20 (9)	21 (10)	20.5 (8)
Cholesterol (mmol/L)	5.33 ± 1.15	4.48 ± 1.04^***^	4.64 ± 1.12^***^
Triglyceride (mmol/L)	1.85 (1.49)	1.16 (0.64)^***^	1.2 (0.9)^***^
HDL-C (mmol/L)	1.01 (0.31)	1.06 (0.36)	1.09 (0.29)^***#^
LDL-C (mmol/L)	3.47 ± 1.05	2.85 ± 0.95^***^	2.87 ± 0.83^***^
HsCRP (mg/L)	1.38 (1.36)	1.22 (2.16)	0.96 (1.65)^*^
FFA (μmol/L)	542 (149.5)	537 (273)	524 (297)
HbA1c (%)	11.18 ± 2.25	9.35 ± 1.52^***^	6.46 ± 0.71^***###^
FPG (mmol/L)	11.32 ± 2.94	5.88 ± 1.08^***^	6.65 ± 1.27^***###^
2hPG (mmol/L)	20.23 ± 3.99	13.9 ± 3.07^***^	11.26 ± 3.42^***###^
HOMA-B	14.62 (22.3)	42.84 (36.57)^***^	59.85 (54.02)^*###^
HOMA-IR	2.94 (2.88)	1.24 (1.15)^***^	2.39 (2.06)^*###^
AUC of glucose	34.39 ± 6.52	22.39 ± 4.5^***^	20.3 ± 4.36^***###^
AUC of insulin	25.49 (18.79)	46.22 (29.78)^***^	53.25 (41.86)^***#^
Matsuda index	88.97 (66.47)	135.72 (88.80)^***^	86.28 (66.94)^###^
ISSI-2	63.45 (58.91)	263.01 (173.82)^***^	250.12 (148.88)^***^

Data were presented as mean ± standard deviation or median (interquartile range). BMI, body mass index; WHR, waist-to-hip ratio; ALT, alanine aminotransferase; AST, aspartate aminotransferase; HDL-C, high-density lipoprotein cholesterol; LDL-C, low-density lipoprotein cholesterol; Hs-CRP, high sensitivity C reactive protein; FFA, free fatty acid; HbA1c, glycated hemoglobin A1C; PG, plasma glucose; INS, insulin; HOMA-IR, homeostasis model assessment of insulin resistance; HOMA-B, homeostasis model assessment of β-cell function; AUC: area under the curve; ISSI-2, Insulin Secretion-Sensitivity Index-2. Compared with baseline, *: P<0.05, **: P<0.01 and ***: P<0.001. Compared with discharge, #: P<0.05 and ###: P<0.001.

We used partial correlation analysis to explore the relationships between circulating DPP-4 activity and various clinical parameters. After adjustment for age, sex, and baseline HbA1c, it demonstrated that the association between circulating DPP-4 activity at baseline and ALT (r=0.36, P<0.001), AST (r=0.36, P<0.001), LDL-C (r=0.24, P=0.02), HOMA-B (r=0.29, P=0.005), HOMA-IR (r=0.27, P=0.011), and the Matsuda index (r=-0.27, P=0.013) reached statistical significance. After SIIT treatment, a correlation was observed between circulating DPP-4 activity and HDL-C (r=0.23, P=0.009) and the 2-hour postprandial glucose (2h-PG) (r=0.28, P=0.013). At the 3-month follow-up, circulating DPP-4 activity was associated with ALT (r=0.38, P=0.001), AST (r=0.50, P<0.001), and HDL-C (r=0.27, P=0.02).

### 3.2 Decreased circulating DPP-4 activity was associated with long-term remission

**Figure 1:**
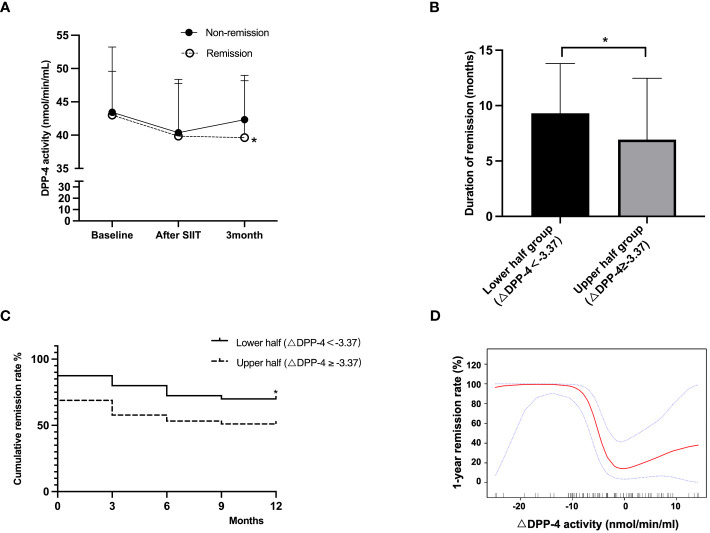
**(A)** Change of circulating DPP-4 activity in the remission and non-remission groups. **(B)** Duration of remission in different △DPP-4 categories. **(C)** Kaplan-Meier survival curves of △DPP-4 groups. **(D)** Smooth curve fitting results between △DPP-4 and 1-year glycemic remission rate. The model was adjusted for age, sex, BMI, cholesterol, FPG, HOMA-B, HOMA-IR, and AUC of insulin at the third month. DPP-4, dipeptidyl peptidase-4; BMI, body mass index; FPG, fasting plasma glucose; HOMA-IR, homeostasis model assessment of insulin resistance; HOMA-B, homeostasis model assessment of β-cell function; AUC: area under the curve. *: P<0.05 between treatment groups.

Of all the participants, 85 (89.5%) completed the one-year follow-up, and 51 (60.0%) remained in remission throughout the follow-up period. Interestingly, despite both the remission and non-remission groups exhibiting similar decreases in circulating DPP-4 activity from baseline to post-SIIT, the circulating DPP-4 activity remained stable in the remission group but rose in the non-remission group at the 3-month follow-up. After adjusting for age, sex, baseline FPG, and TG, the least squares mean of circulating DPP-4 activity were 39.63 ± 8.53 nmol/L and 42.34 ± 6.64 nmol/L in the remission and non-remission groups, respectively, P = 0.032; Thus, the remission group exhibited a more pronounced decrease in circulating DPP-4 activity from baseline to 3 months (△DPP-4, least squares means -3.39 ± 8.90 in the remission group compared to -1.10 ± 8.95 in the non-remission group, P = 0.035) (**Figure 1A**). In addition, patients in the remission group had shorter estimated diabetes course, lower BMI, lower cholesterol and triglycerides, better blood glucose control, better islet function, and better insulin sensitivity 3 months after discharge (**Supplementary Table S1**).

To investigate the association of △DPP-4 with 1-year remission, participants were divided into two groups based on the median of △DPP-4, with the lower half characterized by △DPP-4 <-3.37 nmol/min/mL and the upper half by △DPP-4 ≥ -3.37 nmol/min/mL. A significantly longer duration of remission was observed in patients in the lower △DPP-4 group compared to those in the upper half (9.3 ± 4.5 vs 6.9 ± 5.5 months, P = 0.041) (**Figure 1B**). Additionally, the Kaplan-Meier survival analysis indicated a higher probability of achieving 1-year remission in the lower △DPP-4 group that with more substantial decrease in DPP-4 activity (70.0% vs. 51.1%, P = 0.041 (**Figure 1C**).

To investigate the potential role of △DPP-4 in predicting one-year remission, we conducted logistic regression analyses using the upper △DPP-4 half group as the reference. Initially, adjustments were made for baseline anthropometric parameters in the basal adjusted model. We found that participants with a △DPP-4<-3.37 nmol/min/mL were associated with an increased probability of achieving 1-year remission (OR 2.828, 95% CI 1.078-7.415, P=0.035). This association remained significant after further adjustment for baseline biochemical measurements, as well as insulin sensitivity and β-cell function indices (baseline adjusted model, OR 3.318, 95% CI 1.138-9.667, P=0.028). In the fully adjusted model, which further adjusted for the changes in BMI, insulin resistance and β-cell function indices from baseline to post-SIIT, the odds ratio (OR) for achieving 1-year remission in patients with a △DPP-4 less than -3.37 nmol/min/mL was 7.939 (95% CI 1.829-34.467, P=0.006, **Table 2**).

**Table 2:** Logistic regression analyses on one-year glycemic remission.

	OR	95%CI	P-value
Basal model			
△DPP-4 <-3.37 nmol/min/mL	2.828	1.078 - 7.415	0.035
Baseline adjustment model			
▵DPP-4 <-3.37 nmol/min/mL	3.318	1.138 - 9.677	0.028
Baseline HOMA-IR	1.53	0.998 - 2.362	0.056
Full adjustment model			
▵DPP-4 <-3.37 nmol/min/mL	7.939	1.829 - 34.467	0.006
Baseline BMI	0.74	0.553 - 0.991	0.043
Baseline HbA1c	1.758	1.022 - 3.025	0.042
▵BMI	0.477	0.242 - 0.941	0.033
▵HOMA-IR	0.586	0.358 - 0.96	0.034

Model 1: adjusted for age + sex + BMI.

Model 2: model 1 + baseline triglyceride, HbA1c, HOMA-B, HOMA-IR and ISSI-2.

Model 3: model 2 + △BMI + △HOMA-B + △HOMA-IR.

DPP-4, dipeptidyl peptidase-4; BMI, body mass index; HbA1c, glycated hemoglobin A1C; HOMA-B, homeostasis model assessment of β-cell function; HOMA-IR, homeostasis model assessment of insulin resistance; ISSI-2, Insulin Secretion-Sensitivity Index-2.

We further employed a smooth curve fitting analysis to investigate the mode of association between △DPP-4 and 1-year remission. As shown in **Figure 1D**, the association between △DPP-4 and 1-year remission was nonlinear, with inflection points of -10.0 and -0.7 nmol/min/mL. Patients whose DPP-4 levels had decreased less than 0.7 nmol/min/mL from baseline at 3 months had a low probability of long-term remission, indicating a threshold effect. When △DPP-4 was between -10.0 and -0.7 nmol/min/mL, the odds of one-year remission significantly increased as the △DPP-4 levels decreased. Each 1 nmol/min/mL increase in △DPP-4 was associated with a 63% decrease in the 1-year remission rate (P <0.001). Conversely, a saturation effect was observed for those with a reduction exceeding 10 nmol/min/mL, with no further increase in the chance of long-term remission. (**Table 3**).

**Table 3:** Threshold and Saturation effect analysis of △DPP-4 on events of 1-year glycemic remission.

	Glycemic remission for 1 year (OR, 95% CI)	P-value
Model I (linear analysis)	0.85 (0.76, 0.95)	0.005
Model II (two-piecewise regression models)		
Inflection point of ▵DPP-4	-10, -0.7	
▵DPP-4 activity (-24.84, -10)	1.04 (0.71, 1.54)	0.835
▵DPP-4 activity (-10, -0.7)	0.37 (0.21, 0.66)	<0.001
▵DPP-4 activity (-0.7, 14.19)	1.08 (0.73, 1.6)	0.704
Log-likelihood ratio test		<0.001

DPP-4, dipeptidyl peptidase-4; OR, odds ratio; CI, confidence interval.

## 4 Discussion

To our knowledge, this study is the first to depict the change in circulating DPP-4 activity following SIIT and its relationship with long-term glycemic outcomes in patients with newly diagnosed with T2D. We found a significant reduction in circulating DPP-4 activity after SIIT, which was correlated with various metabolic markers. Crucially, patients with a greater reduction in circulating DPP-4 activity at 3 months post-SIIT had a greater likelihood of achieving one-year remission. The relationship between △DPP-4 and remission is non-linear: the likelihood of remission is low for △DPP-4 above -0.7 nmol/min/mL, but as △DPP-4 decreases below this level, the likelihood of remission increases, reaching a plateau at -10 nmol/min/mL. These data suggest that the changes in circulating DPP-4 activity may serve as a potential predictive marker for SIIT treatment response in newly diagnosed T2D patients.

Although the origin of circulating DPP-4 varies across studies, a substantial body of research suggests a significant contribution from adipose and liver tissues (26, 27). Elevated levels of circulating DPP-4 have been observed in obese patients (28). In human adipose tissue, preadipocytes, adipocytes, and macrophages can all release free DPP-4. Moreover, visceral adipose tissue has higher DPP-4 expression levels compared with subcutaneous adipose tissue (26). The concentration of circulating DPP-4 is associated with both visceral adipose tissue volume and adipocyte size, and increases concomitantly with the obesity progression, the development of insulin resistance, and the worsening of glucose metabolic abnormalities (26). In addition, circulating DPP-4 levels have been shown to increase in patients with non-alcoholic fatty liver disease, and are positively correlated with liver enzymes (29). Rodent studies indicated that hepatic overexpression of DPP-4 contributes to elevated plasma DPP-4 levels, mediating the crosstalk between liver and adipose tissue by activating macrophages and triggering inflammation (30, 31). Conversely, selective suppression of liver DPP-4 expression in obese mouse models resulted in decreased plasma DPP-4 activity levels (27). Circulating DPP-4 is increasingly recognized as a novel adipokine that plays a role in the modulation of insulin sensitivity. This understanding is consistent with the correlation analysis results from our study, which demonstrates that circulating DPP-4 activity is associated with several metabolic indicators, including liver enzymes, lipid profiles, blood glucose levels, and insulin sensitivity indices.

Given the close relationship between circulating DPP-4 and insulin resistance, it is strongly associated with the onset and development of T2D, as confirmed in cohort studies (21). The shedding of DPP-4 from the cell surface increases in high glucose conditions (32), which may contribute to the increased circulating DPP-4 observed in patients with type 2 diabetes. Several treatments for hyperglycemia, including SGLT-2 inhibitors (30), metformin (20), calorie restriction (33), and bariatric surgery (29), can reduce circulating DPP-4 levels. However, there is relatively little research on the role of circulating DPP-4 activity as an indicator of treatment response.

The mechanism by which △DPP-4 predicts remission remains unclarified, but emerging insights into long-term remission of type 2 diabetes in recent years may provide clues. The “Twin Cycle Hypothesis” (34) posits that excessive calorie intake results in hepatic fat accumulation, triggering lipid overflow to the pancreas. The consequent ectopic pancreatic fat deposition impairs β-cell function. To achieve long-term remission, this vicious cycle needs to be disrupted. SIIT works primarily by mitigating glucotoxicity-induced β-cell inhibition, promoting β-cell redifferentiation. However, following the completion of SIIT, the maintenance of treatment response (i.e., long-term remission) relies on the continuous intervention of the aforementioned ‘Twin Cycle’ mechanisms, particularly the strict adherence to lifestyle interventions and the alleviation of fat deposition. It’s reported to reduce ectopic lipid accumulation in skeletal muscle (23) and decrease the visceral to subcutaneous fat ratio (35). Our long-term cohort study identified that a positive attitude toward disease management and commitment to self-care practices are pivotal in reducing insulin resistance and ensuring long-term remission. Given the strong linkage between circulating DPP-4, adipose inflammation, and NAFLD, △DPP-4 may reflect certain key mechanisms of the “Twin Cycle “, suggesting the potential for long-term remission post-SIIT. However, further research is needed to validate this hypothesis.

We did not measure the levels of GLP-1in this study. Huang et al. reported a slight increase in fasting GLP-1 levels only in obese patients after intensive treatment (13). Choi H, et al. reported that GLP-1 response did not change significantly after 4 weeks of SIIT (12). These studies suggest that changes in GLP-1 levels are unlikely to be the main mechanism for long-term remission after SIIT. Moreover, circulating DPP-4 activity only contributes to a small portion of GLP-1 degradation. Instead, endothelial cells and DPP-4 bone marrow-derived cells are major sites where incretin cleavage occurs (36). Therefore, the predictive role of △DPP-4 for treatment response may not be closely related to the traditional incretin effect. Another limitation of this study was that only fasting circulating DPP-4 was measured. However, circulating DPP-4 activity did not change significantly during OGTT in the literature (37). Thus, this limitation is unlikely to affect the reliability of our conclusion.

In summary, circulating DPP-4 activity significantly decreases after short-term intensive insulin therapy. The change in circulating DPP-4 levels at 3 months after cessation of SIIT compared to baseline can be used to predict the long-term glycemic outcomes of patients and for individualized long-term management. Further research is needed to explore the role and mechanism of DPP-4 in the progression and remission of diabetes.

## Data availability statement

The original contributions presented in the study are included in the article/**Supplementary Material**. Further inquiries can be directed to the corresponding authors.

## Ethics statement

The research ethics committee of Sun Yat-Sen University approved the study. The studies were conducted in accordance with the local legislation and institutional requirements. The participants provided their written informed consent to participate in this study.

## Author contributions

JC: Conceptualization, Formal Analysis, Investigation, Writing – original draft. PD: Data curation, Formal Analysis, Investigation, Writing – original draft. WK: Data curation, Writing – review and editing. XW: Funding acquisition, Writing – review and editing. JL: Formal Analysis, Writing – review and editing. LX: Methodology, Writing – review and editing. HX: Project administration, Writing – review and editing. YL: Funding acquisition, Resources, Writing – review and editing. LL: Conceptualization, Funding acquisition, Supervision, Writing – review and editing.
