# Supplementary material for: Decreased circulating dipeptidyl peptidase-4 activity after short-term intensive insulin therapy predicts clinical outcomes in patients with newly diagnosed type 2 diabetes
Source: Front Endocrinol (Lausanne). 2024 Feb 27;15:1352002. doi: 10.3389/fendo.2024.1352002 (PMC10929261; doi:10.3389/fendo.2024.1352002)
Supplement: Supplementary file 1 [file Table_1.docx]

**TABLE S1** - Comparison of clinical characteristic between remission and non-remission groups.

|  | Remission (n = 51) | | | Non-remission (n = 34) | | |  |  |  |
| --- | --- | --- | --- | --- | --- | --- | --- | --- | --- |
|  | Baseline | After SIIT | 3-month | Baseline | After SIIT | 3-month | P-value^1)^ | P-value^2)^ | P-value^3)^ |
| Gender (F/M) | 42/9 |  |  | 27/7 |  |  |  |  |  |
| Age (years) | 47.45 ± 11.8 |  |  | 45.21±10.44 |  |  | 0.371 |  |  |
| Diabetes duration(months) | 1(1.5) |  |  | 2.5(6) |  |  | 0.001** |  |  |
| Body weight (kg) | 72.37 ± 11.01 | 70.79 ± 11.1 | 67.78 ± 10.34 | 74.26 ± 13.2 | 73.48 ± 12.85 | 72.82 ± 12.84 | 0.494 | 0.308 | 0.054 |
| BMI (kg/m2) | 25.65 ± 2.67 | 25.08 ± 2.82 | 24.11 ± 2.81 | 26.3 ± 3.13 | 26.02 ± 2.95 | 25.85 ± 2.99 | 0.326 | 0.144 | 0.009** |
| Waist circumference (cm) | 91.93 ± 8.25 | 90.78 ± 8.45 | 86.42 ± 7.92 | 92.12 ± 8.42 | 91.59 ± 8.4 | 90.39 ± 6.03 | 0.920 | 0.668 | 0.018* |
| WHR | 0.93 ± 0.06 | 0.94 ± 0.06 | 0.9 ± 0.06 | 0.92 ± 0.05 | 0.92 ± 0.05 | 0.92 ± 0.04 | 0.397 | 0.246 | 0.332 |
| ALT (U/L) | 23(17) | 21(14.5) | 20(10.25) | 24(22) | 26(18.5) | 24(20.25) | 0.394 | 0.238 | 0.056 |
| AST (U/L) | 19(8) | 21(8) | 19(7) | 20.5(6.5) | 23(11) | 23(9.75) | 0.285 | 0.131 | 0.022* |
| Cholesterol (mmol/L) | 5.25 ± 1.06 | 4.32 ± 1.04 | 4.33 ± 0.89 | 5.31 ± 1.26 | 4.67 ± 1.02 | 5.12 ± 1.26 | 0.792 | 0.14 | 0.001** |
| Triglyceride (mmol/L) | 1.75(1.41) | 1.02(0.47) | 1.01(0.66) | 1.97(1.58) | 1.11(1.04) | 1.37(1.37) | 0.201 | 0.437 | ＜0.001*** |
| HDL-C (mmol/L) | 1.02(0.31) | 1.03(0.29) | 1.1(0.25) | 0.99(0.28) | 1.06(0.39) | 1.09(0.37) | 0.66 | 0.894 | 0.438 |
| LDL-C (mmol/L) | 3.52 ± 1.15 | 2.73 ± 0.84 | 2.72 ± 0.76 | 3.38 ± 0.88 | 3 ± 0.89 | 3.09 ± 0.88 | 0.558 | 0.158 | 0.05 |
| HsCRP (mg/L) | 1.38(1.01) | 1.25(2.41) | 0.94(1.67) | 1.66(1.49) | 0.95(2.12) | 1.01(1.65) | 0.645 | 0.13 | 0.393 |
| FFA (μmol/L) | 499(193) | 492(272.5) | 474(378.25) | 582(214) | 579.5(270.25) | 533(222) | 0.086 | 0.065 | 0.19 |
| HbA1c (%) | 11.43 ± 2.5 | 9.42 ± 1.62 | 6.13 ± 0.44 | 10.88 ± 2.03 | 9.32 ± 1.49 | 6.97 ± 0.75 | 0.299 | 0.776 | ＜0.001*** |
| FPG (mmol/L) | 11.44 ± 3.46 | 5.92 ± 1.09 | 6.2 ± 0.87 | 11.21 ± 2.34 | 5.85 ± 1.13 | 7.35 ± 1.49 | 0.74 | 0.77 | ＜0.001*** |
| 2hPG (mmol/L) | 20.46 ± 4.54 | 13.6 ± 2.97 | 10.19 ± 2.67 | 19.97 ± 3.15 | 14.14 ± 3.38 | 12.9 ± 3.82 | 0.584 | 0.443 | ＜0.001*** |
| HOMA-B | 15.21(22.82) | 51.29(39.36) | 61.12(53.67) | 13.09(17.8) | 34.64(36.3) | 45.03(55) | 0.352 | 0.115 | 0.436 |
| HOMA-IR | 3.23(2.59) | 1.38(1.22) | 2.09(1.5) | 2.55(3.29) | 1.04(0.98) | 3.29(2.99) | 0.51 | 0.102 | 0.002** |
| AUC of glucose | 34.88 ± 7.26 | 22.35 ± 4.51 | 19.21 ± 3.48 | 33.94 ± 5.66 | 22.6 ± 4.68 | 21.99 ± 5.06 | 0.532 | 0.809 | 0.004** |
| AUC of insulin | 27.15(27.12) | 48.24(29.58) | 53(44.25) | 22.9(16.27) | 42.55(26.65) | 53.6(32.57) | 0.426 | 0.278 | 0.718 |
| Matsuda index | 88.31(66.7) | 130.24(86.98) | 103.97(78.7) | 110.77(74.3) | 143.61(96.13) | 78.34(68.58) | 0.317 | 0.233 | 0.037* |
| ISSI-2 | 62.26(68.4) | 260(169.84) | 281.77(120.37) | 61.65(56.58) | 311.38(189.46) | 182.37(117.48) | 0.815 | 0.673 | ＜0.001*** |
| DPP-4 activity (nmol/min/mL) | 43.03 ± 6.56 | 39.86 ± 7.92 | 39.63 ± 8.53 | 43.44 ± 9.81 | 40.36 ± 8.01 | 42.34 ± 6.64 | 0.830 | 0.776 | 0.051 |
| Data were presented as mean ± standard deviation or median (interquartile range). 1)Differences between remission and non-remission groups at baseline; 2)Differences between remission and non-remission groups after SIIT; 3)Differences between remission and non-remission groups at 3-month. *p < 0.05; **p < 0.01; ***p < 0.001. | | | | | | | | | |
